# Varenicline Effects on Smoking, Cognition, and Psychiatric Symptoms in Schizophrenia: A Double-Blind Randomized Trial

**DOI:** 10.1371/journal.pone.0143490

**Published:** 2016-01-05

**Authors:** Robert C. Smith, Revital Amiaz, Tian-Mei Si, Lawrence Maayan, Hua Jin, Sylvia Boules, Henry Sershen, Chunbo Li, Juanjuan Ren, Yanhong Liu, Mary Youseff, Abel Lajtha, Alessandro Guidotti, Mark Weiser, John M. Davis

**Affiliations:** 1 Nathan S. Kline Institute for Psychiatric Research, Orangeburg, New York, United States of America; 2 NYU Langone Medical Center, Department of Psychiatry, New York, New York, United States of America; 3 Chaim Sheba Medical Center, Ramat-Gan, Israel; 4 Sackler School of Medicine, Tel Aviv, Israel; 5 Peking University Institute of Mental Health, The Key Laboratory for Mental Health, Ministry of Health, Beijing, China; 6 Psychiatric Institute University of Illinois, Chicago, Illinois, United States of America; 7 University of California San Diego, Department of Psychiatry, San Diego, California, United States of America; 8 VA San Diego Healthcare System, San Diego, California, United States of America; 9 Shanghai Key Laboratory of Psychotic Disorders, Shanghai Mental Health Center, Shanghai Jiao Tong University School of Medicine, Shanghai, China; 10 Albany Medical Center, Albany, New York, United States of America; Erasmus University Rotterdam, NETHERLANDS

## Abstract

Schizophrenic patients have a high rate of smoking and cognitive deficits which may be related to a decreased number or responsiveness of nicotinic receptors in their brains. Varenicline is a partial nicotinic agonist which is effective as an antismoking drug in cigarette smokers, although concerns have been raised about potential psychiatric side-effects. We conducted a double-blind placebo controlled study in 87 schizophrenic smokers to evaluate the effects of varenicline (2 mg/day) on measures of smoking, cognition, psychiatric symptoms, and side-effects in schizophrenic patients who were cigarette smokers. Varenicline significantly decreased cotinine levels (P<0.001), and other objective and subjective measures of smoking (P < .01), and responses on a smoking urges scale (P = .02), more than placebo. Varenicline did not improve scores on a cognitive battery designed to test the effect of drugs on cognitive performance in schizophrenia (the MATRICS battery), either in overall MATRICS battery Composite or individual Domain scores, more than placebo. There were no significant differences between varenicline vs. placebo effects on total symptom scores on psychiatric rating scales, PANSS, SANS, or Calgary Depression scales, and there were no significant drug effects in any of these scales sub-scores when we used Benjamin-Hochberg corrected significance levels (α = .05). Varenicline patients did not show greater side-effects than placebo treated patients at any time point when controlled for baseline side-effect scores. Our study supports the use of varenicline as a safe drug for smoking reduction in schizophrenia but not as a cognitive enhancer.

***Trial Registration*:** ClinicalTrials.gov 00802919

## Introduction

Varenicline is an FDA approved drug which has been shown to be efficacious as an anti-smoking treatment in non-psychotic smokers [1, 2]. It is also a high affinity partial nicotinic agonist at the α_2_β_4_ nicotinic receptor, and a full agonist at neuronal α_7_ nicotinic receptor [3]. Schizophrenic patients show a high rate of cigarette smoking and cognitive deficits which may be related to defects in their nicotinic receptors. Several studies have shown reduced numbers of α-bungarotoxin binding sites in the hippocampus of schizophrenic patients, indicating a reduction in the α_7_ neuronal nicotinic receptor numbers [4, 5],and differences have been reported in the promoter region of the α-7 nicotinic receptor gene in schizophrenia [6]. Cigarette smoking may transiently improve a psychophysiological measure related to sensory gating deficits in schizophrenia [7, 8], and cigarette smoking or nicotine administration may also improve their performance on cognitive tests [9–11], [12–14] [15, 16]. Our preliminary open study of varenicline's effects in schizophrenics showed some cognitive improvement in RBANS scores [17] and a lack of negative psychiatric effects. However, early reports, from Med Watch submissions to the FDA, suggested that varenicline might have psychiatric side-effects, including increases in depression, suicide or psychosis in vulnerable patients. To investigate these multiple issues a double-blind placebo controlled study of the effects of standard clinically used doses of varenicline was conducted on measures of smoking, cognition, psychopathology and side effects in patients with schizophrenia who were cigarette smokers, using several objective and subjective measures. We hypothesized that varenicline would improve cognition and reduce smoking in patients with schizophrenia. Subsequent to the initiation of this study, two other double -blind studies were published on the effects of varenicline on cognition and symptoms in schizophrenia [18, 19] and these are compared in the discussion section.

## Method

### Subjects, Design, and Sites

This was an 8-week double-blind randomized parallel group design study (at 4 sites -2 US, 1 Israel, and 1 China) of varenicline and matched placebo (supplied by Pfizer) in patients with a DSM-IV diagnosis of schizophrenia or schizoaffective psychosis, who were treated with antipsychotic medication, were cigarette smokers and had RBANS scores < 90. The study was conducted between February 2009 and January 2013. Although an 8 week trial was deemed sufficient and preferable by the grant funding agency (Stanley), at some sites smoking and symptom measures were continued for 12 weeks of treatment because other studies focused on smoking cessation with varenicline had used 12 week trials. Patients were outpatients (65) or inpatients (22) and were either current cigarette smokers (≥ 6 cigarettes/day) or, if hospitalized in a US non-smoking hospital facility, were recent chronic smokers before hospitalization who had violated non-smoking rules on several occasions and continued to smoke occasional cigarettes while hospitalized even through the hospital tried to enforce a non-smoking policy. (The minimal smoking level of 6 cigarettes/day was chosen as the entry criteria for current smokers because the recent increase in cost of cigarettes at U.S. sites had an impact on reducing availability of cigarettes for schizophrenic patients.) Patients were willing to participate in a trial of a drug which might reduce their smoking and improve cognition, but not necessarily to quit smoking, and there was no "quit date" as part of the study design or smoking counseling procedures. Subjects were excluded if they were currently taking anti-smoking drugs (bupropion, varenicline, nicotine) had a total PANSS score >90, had a PANSS depression item score >5, or had a Calgary depression scale score >20. (For detailed exclusion and inclusion criteria see S1 File.) All subjects received brief (5–10 minute) cigarette smoking prevention counseling at each weekly study visit using a structured program which provided different written information supplemented by verbal counseling at weekly visits. (Procedures were modified and shortened from a manual provided by Eden Evins [20, 21]). Patient's antipsychotic and other psychotropic medications remained stable during the course of the study period. The study was registered at clinicaltrials.gov NCT# **00802919**, and a consort check list is attached (S1 CONSORT Checklist).

### Ethics Statement

Patients signed informed consent for a protocol approved by the IRB for each site (Nathan S. Kline Institute for Psychiatric Research, Chaim Sheba Medical Center, and Institute of Mental Health at Peking University)(S1 Protocol).

### Study Drug Doses and Administration

Patients were randomly assigned (1:1) to receive either varenicline or matched placebo tablets. Each site had its own computer generated randomization table, and patients were randomized in groups of 4 (2 placebo 2 varenicline in each group) (see S1 File for further details). Varenicline was administered in a dose of 0.5 mg—1.0 mg/day for the first week, and 2.0 mg/day (two 1 mg tablets) for the remainder of the study period. For outpatients and inpatients drug supply was given out in a weekly bottle, which was returned the following week when they picked up their next week’s supply. Any remaining pills were counted and the reason for any remaining pills was recorded. For most outpatients who lived in community residences, at the U.S. and foreign sites, medication bottles were given to the nurse at the community residence who administered the medication to the subjects at set times of medication dispensation at their facility. Patients who lived independently or with their families administered the medication themselves from each weekly supply bottle.

### Evaluations

Subjects were evaluated for smoking by: a)self-report number of cigarettes smoked in last week, b) Breathalyzer CO levels weekly, c)nicotine and cotinine levels in plasma (baseline and then monthly), d) smoking urges—QSU smoking urges scale weekly [22], and the Cigarette Dependence Scale (baseline and end of study)[23]. Details of methods to determine breathalyzer CO levels and nicotine and cotinine are described in our previous publications [9, 11], and the methods for nicotine and cotinine determination at the Chinese site is described in (S1 File). They were evaluated for psychopathology with the PANSS scale (Positive and Negative Symptom Scale) (baseline and once every 4 weeks)[24], SANS (Scale for Assessment of Negative Symptoms) (baseline and 8 weeks)[25], and Calgary Depression Scale (weekly)[26]. The SANS scale was truncated to exclude all the attention items (because we had found poor response in our population to these items and other researchers had found that it did not reflect social inattentiveness [27]). For the main psychopathology (PANSS, SANS) scales clinically experienced raters (who had achieved ICC's of ≥ 0.80 on total scores) performed the ratings, and the same rater rated the patient on all occasions. However, there was no inter-site rater training or comparisons. Patients were evaluated for cognition at baseline with the RBANS cognitive scale (Repeatable Battery For The Assessment Of Neuropsychological Status) [28], and with the MATRICS consensus battery (MCCB)[29] at baseline and 8 weeks (or end of study if terminated before 8 weeks), without the social cognition module. Side-effects were evaluated with a side-effect checklist (baseline, week 2, week 4, week 8, week 12) (supplied by researchers at Maryland Psychiatric Research Institute and cited in their prior studies) [30] and also with a free form inquiry weekly.

### Statistical Analysis

For each measure subjects were included for analysis if they had at least one post-baseline value entered for the variable. The primary analysis was carried out on data from the 8-week double-blind study, and supplementary analysis was carried out on the sub-set of patients who completed an additional 4 weeks of the study. The main analysis for most variables was a mixed linear model repeated measures analysis of covariance (baseline value as covariate) using the SAS mixed procedure program with either unstructured or autoregressive (ar1) correlation structure, with Drug and *Site* as factors and Time *the* repeated measure. For measures with only baseline and 8 week (or end) values, we utilized SPSS univariate ANCOVA. Supplementary non-parametric analysis was performed on variables which deviated substantially form normality (see S1 File for further details on methods). For those variables which had significant effects in the mixed model analysis with missing data, a similar analysis was performed using traditional LOCF for missing data to examine the robustness of the results. Results are presented using traditional significant levels (i.e. uncorrected for multiple comparisons), and for some significance levels corrected for multiple comparison by the Benjamini-Hochberg (BH) procedure [31]. Effects sizes were calculated using Cohen’s d and/or partial eta squared (from the SPSS ANOVAs).

## Results

### Subject Participation

**Fig 1:**
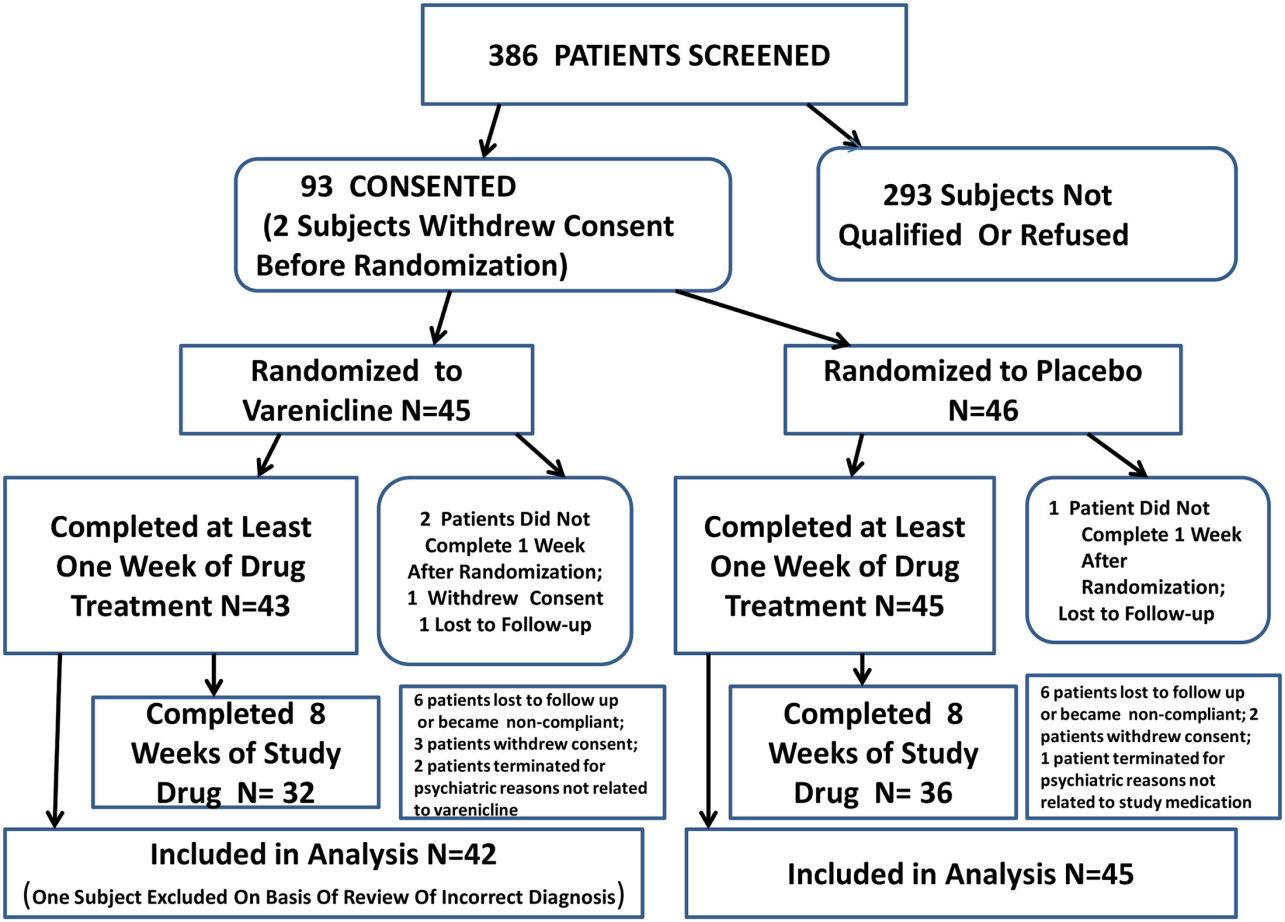
Flow chart of disposition of patients through the study. Number of patients who were used for each major outcome measures in main analysis: **Cognition**: MATRICS battery Composite Score N = 54, Varenicline = 25, Placebo = 29; for number of subjects analyzed for individual Domain scores on MATRICS see Table 3 and Fig 3. **Smoking Measures**-Cigarettes Smoked and Breathalyzer CO N = 87, Varenicline = 42, Placebo = 45; Nicotine and Cotinine N = 70, Varenicline = 34, Placebo = 36; Smoking Urges QSU N = 85, varenicline = 40, Placebo = 45; Psychopathology Measures—PANSS Scale N = 77, Varenicline = 38, Placebo = 39; SANS Scale n = 64, Varenicline = 30, Placebo = 34; Calgary Depression Scale N = 74, Varenicline = 36, Placebo = 38.

Overall, 93 subjects were consented, 91 were randomized, and 87 provided valuable data (S3 File) on at least one outcome measure. The patient disposition flow chart is shown in Fig 1.

### Baseline Characteristics

Table 1 shows the characteristics of subjects in the active and placebo groups who were utilized for analysis of one or more outcome measures. There were no significant differences in any baseline characteristics of the subjects randomized to varenicline or placebo (Table 1). The subjects were predominantly male patients with chronic schizophrenia (76 M, 13F), treated for many years with antipsychotics (26% on clozapine, and 37% on multiple antipsychotics) and many were also treated with accessory medication. These patients showed moderate to severe cognitive deficits compared to norms in other published studies. The mean RBANS scores were low in both groups (mean 66–67) which is about 9% of the normal standardization sample [28]. The MCCB overall composite score in both groups (mean 17–18) was well below the normative sample for the MCCB battery in this age group by Kern and associates [32] or schizophrenic patients in this age group reported by Rajji et al. [33]. All MCCB domain scores were also similarly low compared to these studies. Patients had low to moderate levels of psychopathology (PANSS mean scores 56–58) without significant current depressive symptoms (mean Calgary Depression scores <2). They were smokers for many years (means 18–23), and most had substantial levels of plasma cotinine, indicating recent moderate to high smoking. (The “0” value in the range for cotinine and cigarettes/day come from a few inpatients in U.S. non-smoking inpatient wards, who were chronic smokers but were not smoking on the day of their baseline assessment.)

**Table 1:** Subject characteristics in varenicline and placebo groups. N = 87 (varenicline N = 42, placebo N = 45) based on subjects who had one post baseline reading on at least one outcome variable. Actual N's for some variables are lower because of missing data on selected subjects.

Subject Characteristics	Placebo	Varenicline	Test For Difference
Age (M±S.D.)	43.6 ± 10.6	46.6 ± 8.9	T = 1.406, DF = 85,P = 0.163
Sex (N)			χ2 = 0.190 P = 0.663
M	39 (85%)	35 (83%)	
F	6 (15%)	7 (16%)	
Race/Ethnicity (N)			χ2 = 0.392, P = 0.950
W	13 (29%)	14 (33%)	
B	17 (38%)	15 (35%)	
H	7 (15%)	5 (11%)	
Ch	8 (18%)	8 (19%)	
Diagnosis (N)			χ2 = 2.052, P = 0.178
S	34 (75%)	28 (66%)	
SA	11 (24%)	14 (33%)	
Outpatient/Inpatient (N)^a^			χ2 = 0.035, P = 0.851
OP	34 (75%)	31 (73%)	
IP	11 (24%)	11 (26%)	
Antipsychotic Type (N)			χ2 = 3.567, P = 0.159
l^st^ Gen	5 (11%)	7 (16%)	
2^nd^ Gen	35 (77%)	25 (59%)	
Combined	5 (11%)	10 (23%)	
On Clozapine (N)			χ2 = 1.047, P = 0.306
Y	14 (31%)	9 (21%)	
N	31 (68%)	33 (78%)	
Antidepressant (N)			χ2 = 1.245, P = 0.265
Y	8 (17%)	4 (9%)	
N	37 (82%)	38 (90%)	
Mood Stabilizer (N)			χ2 = 0.200, P = 0.654
Y	13 (28%)	14 (33%)	
N	32 (71%)	28 (66%)	
Benzodiazepine (N)			χ2 = 0.187, P = 0.666
Y	10 (22%)	11 (26%)	
N	35 (77%)	31 (73%)	
Anti-parkinsonian Medication(N)			χ2 = 0.198, P = 0.653
Y	5 (11%)	6 (14%)	
N	40 (88%)	36 (85%)	
PANSS Total (M±S.D.)	58.8 ± 15.7	56.2 ± 14.9	T = 0.758, DF = 85, P = 0.451
Calgary Depression Scale Total (M±S.D.)	1.9 ± 2.8	1.3 ± 2.6	NP Z = 1.312, P = 0.190
RBANS Total (M±S.D.)	65.9 ± 13.1	67.5 ± 13.2	T = .570, DF = 85, P = 0.570
MATRICS Overall Composite (M±S.D.)	17.4 ± 12.3	17.9 ± 11.1	T = 0.71, DF = 73, P = 0.865
Cigarette Dépendance Scale Total (M±S.D.)	30.7 ± 9.6	30.2 ± 7.2	T^U^ = 0.277, DF = 77.3, P = 0.782
Cigarettes Smoked/day (M±S.D.) (range)	17.1 ± 13.3 (0–60)	18.0 ± 19.0 (0–100)	T = 0.260, DF = 85,P = 0.796
Years Smoker^b^ (M±S.D.)	18.0 ± 11.1	22.8 ± 11.6	T = 1.538, DF = 57, P = 0.130
Serum Cotinine (ng/ml) (M±S.D.) (range)	242.1 ± 161.9 (0.0–668.5)	251.3 ± 171.9 (O.O—628.1)	T = 0.335, DF = 82, P = 0.738

^**a**^ Most of the inpatients came from a site (China) where inpatients were allowed to smoke cigarettes. The 6 inpatients at the U.S. sites were in smoke free facilities, but had been regular cigarette smokers before hospitalization and had violated no-smoking rules during hospitalization although they were not regularly smoking cigarettes at time of entry into the study.

^**b**^ Years of smoking data collected on selected subjects, varenicline N = 28, placebo N = 27. S = Schizophrenia. SA = Schizoaffective. W = White, B = Black or African American, H = Hispanic, Ch = Chinese Han Gen = Generation.(N) = Number of subjects (M± S.D) = Mean ± Standard Deviation. χ2 = CHI Square statistic (df for χ2 = 1). T = 2 sample t-test, equal variances. T^U^ = 2-sample t-test unequal variances. NP = non-parametric test (Mann-Whitney U).

### Drug Ingestion

In their weekly reports patients who were continuing in the study reported that they had ingested all the medication in the bottle for that week. Almost all bottles were returned empty, except for patients who decided to terminate their participation in the study during a specific week. In the U.S. outpatients, only 3 patients who continued in the study returned pill bottles at the end of one or two of the specific weeks which were not empty (1 patient 1 pill, 1 patient 2 pills, 1 patients 4 pills), which they explained by intercurrent events (out of facility or ill for one or two days). No patient reported that they stopped or reduced taking medication because of perceived study drug side-effects.

### Effects on Cigarette Smoking

**Fig 2:**
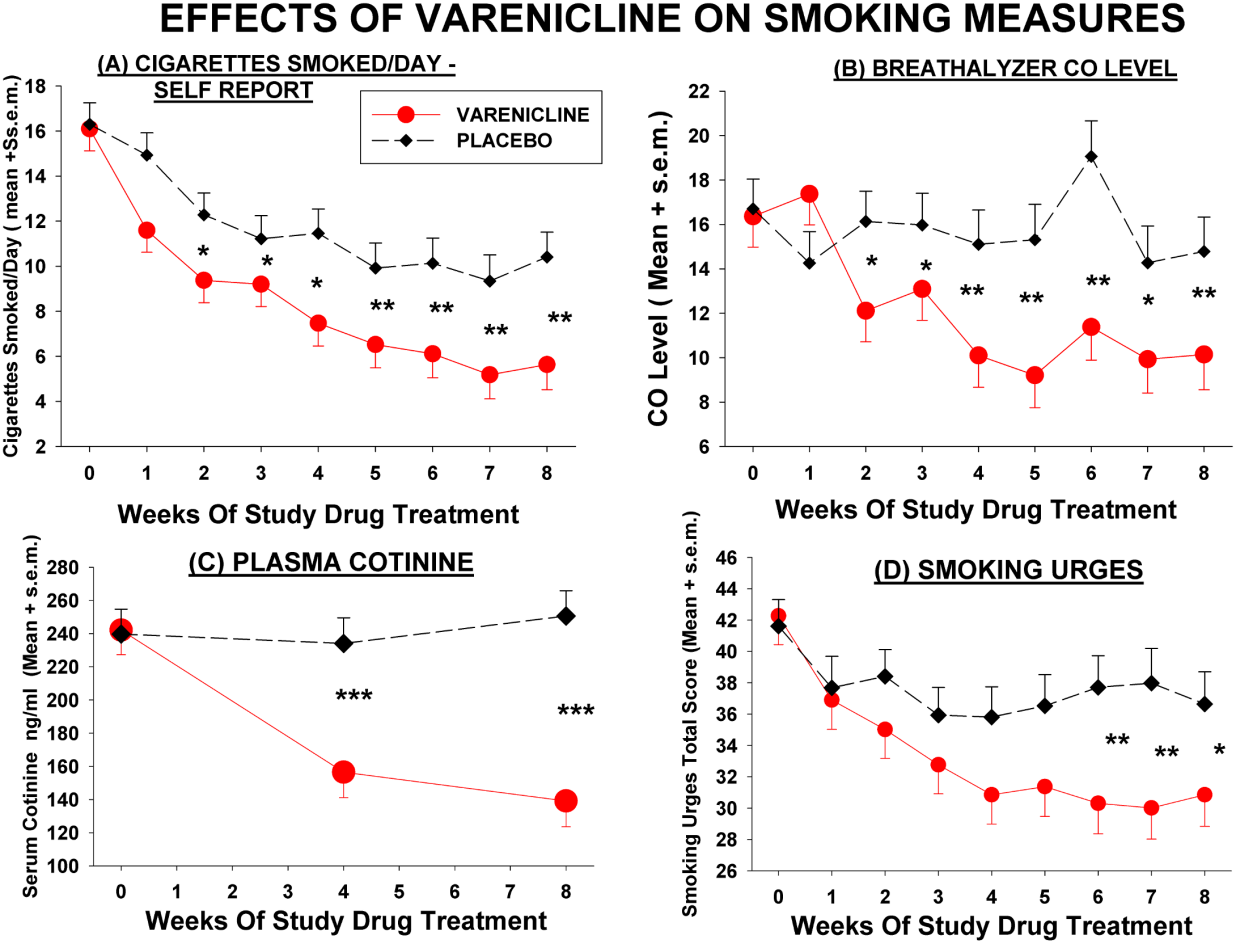
Effects of varenicline and placebo on measures related to cigarette smoking. Each value represents model adjusted least square mean score ± s.e.m. for that week, from mixed model ANCOVA. Significance of difference between varenicline and placebo means at specific time point (t-test): * P < .05, **, P < .01, *** P < .001. For Cigarette and CO values, statistics were calculated from analyses using square-root transformed values, which better approximated a normal distribution. Overall *Drug Effect* from mixed model ANCOVA: *(A)* Cigarettes Smoked/Day (N = 87, Varenicline = 42, Placebo = 45) *Drug Effect* F = 14.88, DF = 1, 81, P = 0.0002; *Drug x Time Effect*: F = 1.67, DF = 8,532, P = 0.1035. *(B*) C0 (N = 87, Varenicline = 42, Placebo = 45*) Drug Effect*: F = 16.27, DF = 1,81, P = 0.0001; *Drug x Time Effect*: F = 2.08 DF = 8,535, P = 0.0357; *(C)* Cotinine (N = 70, varenicline = 34, Placebo = 36) *Drug Effect*: F = 16.30, DF = 1,64, P = 0.0001; *Drug X Time Effect*: F = 8.81, DF = 2,112, P = .00003,; *(D)* Smoking Urges (QSU) (N = 85, varenicline = 40, Placebo = 45) *Drug Effect*: F = 7.20, DF = 1,79, P = 0.0089; *Drug X Time Effect*: F = 0.86, DF = 8,518, P = 0.5502.

Varenicline significantly reduced smoking and cigarette craving on all the objective and self-report measures, except the cigarette dependence scale, in patients with schizophrenia (Fig 2). Further analysis of covariance of difference scores from baseline (Table 2) showed strong drug effects on number of cigarettes smoked (P = 0.010), CO levels (P = 0.003), plasma nicotine (P = 0.045), plasma cotinine (P<0.001), and total scores on QSU brief smoking urges scale (P = 0.022). Most of the drug effects of varenicline on smoking measures were seen by week 2 or week 4 of study drug treatment, and the magnitude of the difference between varenicline and placebo groups showed a trend for increase on some measures in subsequent weeks. However the effect of varenicline on smoking urges only became statistically significant at week 5 and plasma nicotine levels were significantly decreased at week 8 but not week 4 of drug treatment. Most of the varenicline effects persisted to week 12 in the sub-sample of patients who continued treatment an additional 4 weeks, but the magnitude of the effects did not increase with continued treatment. The decrease in CO levels with varenicline remained significant in non-parametric analysis (Mann-Whitney U) in observed cases analyses at each week of drug treatment. LOCF analysis of covariance showed the same general results as the mixed-model analyses. Effect size for varenicline, based on *Cohen's d*, calculated from adjusted means or mean differences at 8 weeks of drug treatment, were moderate to large: for cotinine 1.06–1.26, for nicotine 0.52, for self reported smoking 0.53–0.55, for breath CO 0.57–0.67, and for smoking urges(QSU) 0.42–0.48. Considering the 6 main analyses (Table 2) of the smoking measures using the main (8 week) study data, BH corrected significance levels showed significant effects of varenicline (at false discovery rate of 0.05) for serum cotinine, CO, self-report of cigarettes smoked, and smoking urges.

**Table 2:** Change from baseline in smoking related measures at selected weeks of treatment after baseline. Each value represents mean ± s.e.m. of model estimated difference score (wk_**i**_-baseline). Difference of mean difference from 0 (no change) for measure for each drug group at specific time point:

Measure And Week Of Study Drug Treatment	Varenicline (Mean ± s.e.m)	Placebo (Mean ± s.e.m.)	T-Test or Contrast at Specific Time Point	Overall *Drug Effect* Full Model (F)	Overall *Drug X Time Effect* Full Model (F)
**Reported Cigarettes Smoked/Day (N = 87)**^a^					
Week 1	-4.05 ± 1.18**	+0.10 ± 1.18	**T = 2.49, DF = 453, P = 0.013**	**F**^**1**^ **= 6.91, DF = 1,81, P = .0.010**	F^1^ = 1.02, DF = 7,453, P = .0.409 ^d^
Week 4	-7.94 ± 1.20***	-3.72 ± 1.24**	**T = 2.46, DF = 453, P = 0.014**		
Week 8	-9.40 ± 1.29***	-4.76 ± 1.29 ***	**T = 2.55, DF = 453,P = 0.011**		
Week 12 (N = 65)	-3.73 ± 1.05***	-1.92 ± 0.89*	T = 1.31, DF = 555, P = 0.190	F^2^ = 3.55 DF = 1,60, P = 0.064	F^2^ = 1.54, DF = 11,555, P = 0.114
**Breathalyzer CO Level (N = 87)**^a^					
Week 1	+1.38 ± 1.50	+1.32 ± 1.53	T = -0.03, DF = 456,P = 0.978	**F**^**1**^ **= 9.45, DF = 1,81, P = 0.003**	F^1^ = 1.57, DF = 7,456, P = 0.143
Week 4	-5.88 ± 1.54***	-0.80 ± 1.66	**T = 2.25, DF = 456, P = 0.025**		
Week 8	-5.87 ± 1.70***	-0.91 ± 1.67	**T = 2.08, DF = 456, P = 0.038**		
Week 12 (N = 65)	-4.20 ± 1.86*	+0.70 ± 1.66	**T = 1.97, DF = 555, P = 0.050**	**F**^**2**^ **= 6.06, DF = 1, 60, P = 0.017**	F^2^ = 1.21, DF = 11,555 P = 0.277
**Plasma Nicotine levels (ng/ml) (N = 70**)^b^					
Week 4	-4.47 ± 1.36**	-1.61 ± 1.38	T = 1.48, DF = 64, P = 0.144	**F**^**1**^ **= 4.18, DF = 1,64, P = 0.045**	F^1^ = 0.013, DF = 1,64, P = .0.723
Week 8	-6.18 ± 1.14**	-2.69 ± 1.17*	**T = 2.17, DF = 64, P = 0.033**		
Week 12 (N = 44)	-4.22 ± 1.94*	+0.39 ± 1.57	T = 1.85, DF = 65, P = .070	**F**^**2**^ **= 6.06, F = 1,40, P = 0.018**	F^2^ = 0.01, DF = 2,65, P = 0.994
**Plasma Cotinine Level (ng/ml) (N = 70)**^b^					
Week 4	-80.79 ± 18.35***	-7.32 ± 18.62	**T = 2.81, DF = 50, P = 0.007**	**F**^**1**^ **= 15.21, DF = 1,64, P<0.001**	F^1^ = 2.52, DF = 1,50, P = 0.119
Week 8	-100.66 ± 18.50***	+9.00 ± 18.29	**T = 4.21, DF = 50, P<0.001**		
Week 12 (N = 44)	-82.84 ± 25.58**	+29.69 ± 20.73	**T = 3.52, DF = 80, P<0.001**	**F**^**2**^ **= 20.44, DF = 1,52, P<0.001**	F^2^ = 0.80, DF = 2,80, P = 0.451
**Smoking Urges (QSU-10) (N = 85)**^a^					
Week 4	-10.63 ± 1.97***	-6.06 ± 2.07**	T = 1.60, DF = 441, P = 0.111	**F**^**1**^ **= 5.54, DF = 1,79, P = 0.022**	F^1^ = 0.57, DF = 7,441, P = 0.781
Week 8	-11.54 ± 2.10***	-5.48 ± 2.23*	**T = 1.98, DF = 441, P = 0.049**		
Week 12 (N = 62)	-9.39 ± 2.71**	-6.45 ± 2.46**	T = 0.80,DF = 552, P = 0.424	F^2^ = 2.30, DF = 1,59, P = 0.135	F^2^ = 0.64, DF = 11,552 P = 0.792
**Cigarette Dependence Scale (N = 57)**^c^					
End Study vs. Baseline	-1.55 ± 1.21	-0.050 ± 1.22	NA	F = 0.764, DF = 1,48, P = .387	NA

* P < .05,

**P < .01,

*** P < .001. Statistical analyses were SAS mixed-model of difference scores with baseline value of the variable (or its appropriate transform) as covariate.

^**a**^ F based on mixed model analyses of difference values from all data in week 1 to week 8 vs. baseline (F^1^)for main sample. (F^2^) from week 1 to week 12 for reduced sample based on patients who were scheduled to complete 12 weeks of study drug treatment with this measure. Only values from selected weeks are shown in table, although all weeks are used in overall model analysis.

^**b**^ F based on analysis from mixed-model of difference scores in week 4 and week 8 vs. baseline for main analysis, and week 4, week 8 and week 12 for reduced sample. Only values for some weeks are shown.

^**c**^ F from Univariate ANCOVA using SPSS GLM with baseline value as covariate, drug and site as fixed factors. *End Point* of assessment was 12 weeks in US and China samples and 8 weeks in Israeli sample. NA = not applicable.

^**d**^ *Drug X Time X Site Effects*. ***Self Report Cigarette Measure***—There were site differences in the drug effect (*drug x time x site* F = 1.83, DF = 45,453, p = 0.0003), with the Israeli site showing the largest estimated active varenicline decrease and one of the US sites showing the smallest varenicline decrease effect. Since the Israeli site did not progress to 12 weeks, this might explain the smaller drug effect in the reduced sample size at 12 weeks. ***Smoking Urges (QSU***)—Although there was not a statistically significant *drug x time x site* effect from the overall analysis, the Israeli site tended to have the greatest effect of active varenicline on decreasing smoking urges. The elimination of the Israeli site from the 12 week sample may explain the lack of significant decreases in smoking urges in varenicline vs. placebo comparison in the 12-week sub-sample.

However, in this group of schizophrenic patients who did not have a goal of quitting smoking or indicate a definite desire to totally quit, few patients actually quit smoking as assessed by traditional smoking cessation end-points. For those patients who continued in the protocol for the full 8 weeks only 7 (22.6%) of the varenicline patients and 4 (11.1%) of the placebo patients reported no cigarette smoking for the 7 days prior (difference between groups χ2 = 1.60, P = 0.206). There was also no difference on an objective measure of no smoking (cotinine <20 ng/ml); 7 of the varenicline patients (24.1%) and 5 of the placebo patients (14.3%) had cotinine levels ≤ 20 ng/ml by 8 weeks (χ2 = 1.01, P = 0.315). Examining the same measures with LOCF analysis did not improve estimated quit rates. There was also no drug effect on the change scores, from baseline to end-point, on the *Cigarette Dependence Scale* (Table 2).

### Cognition

**Fig 3:**
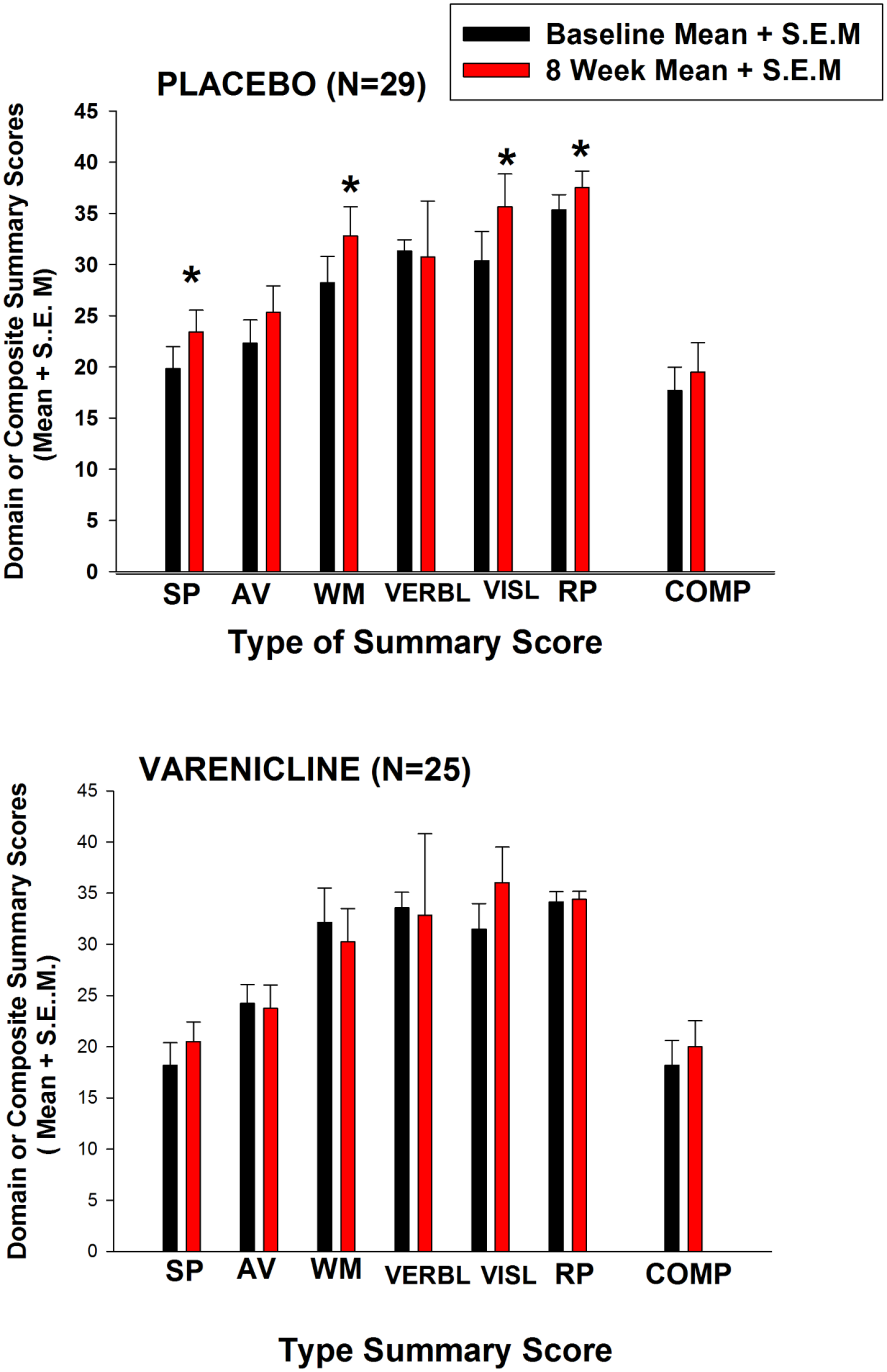
Comparison of MATRICS Battery Domain and Overall Composite Scores In varenicline- and placebo- treated patients. N's are shown in figure. Each value represents mean ± s.e.m. of Domain or Composite scores of subjects who had complete values on all Domain scores at baseline and week 8. Mean scores are *not* adjusted for baseline covariate value or site effects. Abbreviations of Domain and Composite scores in figure: SP = speed of processing, AV = attention-vigilance, WM = working memory, VERBL = verbal learning, VISL = visual learning, RP = reasoning-problem solving, COMP = overall composite. Significance of difference for specific Domain or Composite score between baseline and 8 week value: *P < .05, by paired t-test.

Varenicline did not improve cognitive function in schizophrenic patients compared to placebo. Adjusted MATRICS difference scores from baseline to 8 weeks were not greater for varenicline on overall Composite score, or any of the Domain summary scores (Table 3). Although there were some significant differences favoring placebo or varenicline in some of the tests with significance levels uncorrected for multiple comparisons, none of the significant drug effects from the ANOVAs remained statistically significant when corrected significance levels were calculated with the BH procedure (either at false discovery rates of 0.05, 0.10, or 0.20). For example, the Domain score of *Reasoning and Problem Solving* placebo treated patients improved significantly more than did varenicline patients with uncorrected significance levels. Additionally, both individual T-scores and raw scores were examined for each test. Although raw scores are not traditionally analyzed, it was not certain that the T-scores were accurate across cultures, because the MATRICS computer program norms for calculating T-scores were based on US standards and separate norms for China and Israel had not been definitely established or added to this version of the computer program. An occasional comparison (e.g. Trial Making test) showed a difference at uncorrected significance levels which did not survive the BH correction (see Table 3). Furthermore, when Domain scores (unadjusted for baseline covariate) were compared for the sub-set of patients who had complete data on all Domain scores at baseline and 8 weeks (Fig 3), the placebo patients showed significant improvement on scores in several Domains, comparing scores from baseline to 8 weeks, while the varenicline treated patients showed no change in any Domain score. Non-parametric tests, run on specific difference scores which showed substantial deviation from a normal distribution, found no difference for either individual T-scores or summary Domain scores between varenicline and placebo groups.

**Table 3:** Change from baseline in MATRICS Battery Scores by week 8 of drug treatment. Each value represents mean ± s.e.m of model estimated difference score (wk_**8**_-baseline). Difference of mean difference from 0 (i.e., no change) for measure for each drug group at specific time point:

Measure	Varenicline (N = 25–32)	Placebo (N = 29–35)	*Drug Effect* Overall Model (F)	*Drug x Site Effect* Overall Model (F)
**OVERALL COMPOSITE AND DOMAIN SUMMARY SCORES**				
OVERALL COMPOSITE Score (N = 54)	-0.19 ± 2.14	+1.67 ± 1.86	F = 0.439, DF = 1,45, P = 0.511	F = 1.234, DF = 3,45, P = .0.308
SPEED OF PROCESSING (N = 66)	+3.03 ±1.52	+4.18 ± 1.56*	F = 0.278, DF = 1,57, P = 0.600	F = 1.659, DF = 3,57, P = 0.186
ATTENTION-VIGILANCE (N = 56)	+2.49 ± 1.99	+4.33 ± 1.95*	F = 0.436, DF = 1,47, P = 0.512	F = 1.716, DF = 3,47, P = 0.176
WORKING MEMORY (N = 67)	+0.95 ± 1.82	+5.29 ± 1.88*	F = 2.736, DF = 1,58, P = .103	F = 0.996, DF = 3,58, P = 0.401
VERBAL LEARNING (N = 67)	+0.94 ± 1.00	+0.01 ± 1.04	F = 0.412, DF = 1, 58, P = 0.524	F = 0.683, DF = 3,58, P = 0.566
VISUAL LEARNING (N = 66)	+4.75 ± 2.26*	+7.86 ± 23.0*	F = 0.939, DF = 1,57, P = 0.337	**F = 4.973, DF = 3,57, P = .004**^a^
REASONING and PROBLEM SOLVING (N = 66)	+0.38 ± 0.78	+2.79 ± 0.81*	**F = 4.585, DF = 1,57, P = 0.037**	F = 0.458, DF = 3,57, P = 0.713
**SPECIFIC TEST T-SCORES**				
Trail Making Test A (N = 67)	+6.29 ± 1.88*	+0.26 ± 1.98	**F = 4.817, DF = 1,58 P = 0.032**	F = 1.971, DF = 3,58, P = 0.128
Hopkins Verbal Learning Test (N = 67)	1.97 ± 1.05	-0.02 ± 1.09	F = 1.703, DF = 1,58, P = 0.197	F = 0.193, DF = 3,58, P = 0.901
Category Fluency (N = 65)	-0.94 ± 1.48	+2.00 ± 1.51	F = 1.921, DF = 1,56, P = 0.171	F = 0.405, DF = 3,56, P = 0.750
**SPECIFIC TEST RAW SCORES**				
Trail making Task A (N = 67)	-12.51 ± 4.15*	-0.213 ± 4.35	**F = 4.155, DF = 1,58, P = 0.046**	F = 1.077, DF = 3,58, P = 0.366
Hopkins Verbal Learning Test (N = 67)	0.99 ± 0.76	0.10 ± 0.78	F = 0.667, DF = 1,58, P = 0.417	F = 0.326, DF = 3,58, P = 0.807
Category Fluency (N = 65)	-1.10 ± 0.71	+0.84 ± 0.73	F = 3.538, DF = 1,58, P = 0.063	F = 0.282, DF = 3,58, P = .0.838

* P<0.05. F from Univariate ANCOVA of difference score (week 8-baseline) using SPSS GLM with baseline value (or its appropriate transform) as covariate, and drug and site as fixed factors. Overall Composite scores could not be computed on all subjects because of missing scores on one or more tests needed for such computation by the MATRICS computer program.

^**a**^ For visual learning Israeli patients had significantly poorer performance after 8 weeks of treatment with varenicline and U.S. patients at one of the two US sites had better performance after varenicline.

Intensity of smoking at baseline, as indicated by the objective measure of nicotine and cotinine levels had a small but significant relationship to improved cognitive performance in the schizophrenic patients who were smokers. For example serum cotinine levels were significantly correlated with MCCB composite score (r = 0.35 P = 0.006) and the domain score of attention-vigilance (r = 0.42, P = 0.001), and these correlations were slightly greater in the U.S. sample. However, there were no significant correlations between change in cotinine levels after eight weeks of study drug treatment and changes in any component of MCCB scores.

### Psychiatric Symptoms

**Fig 4:**
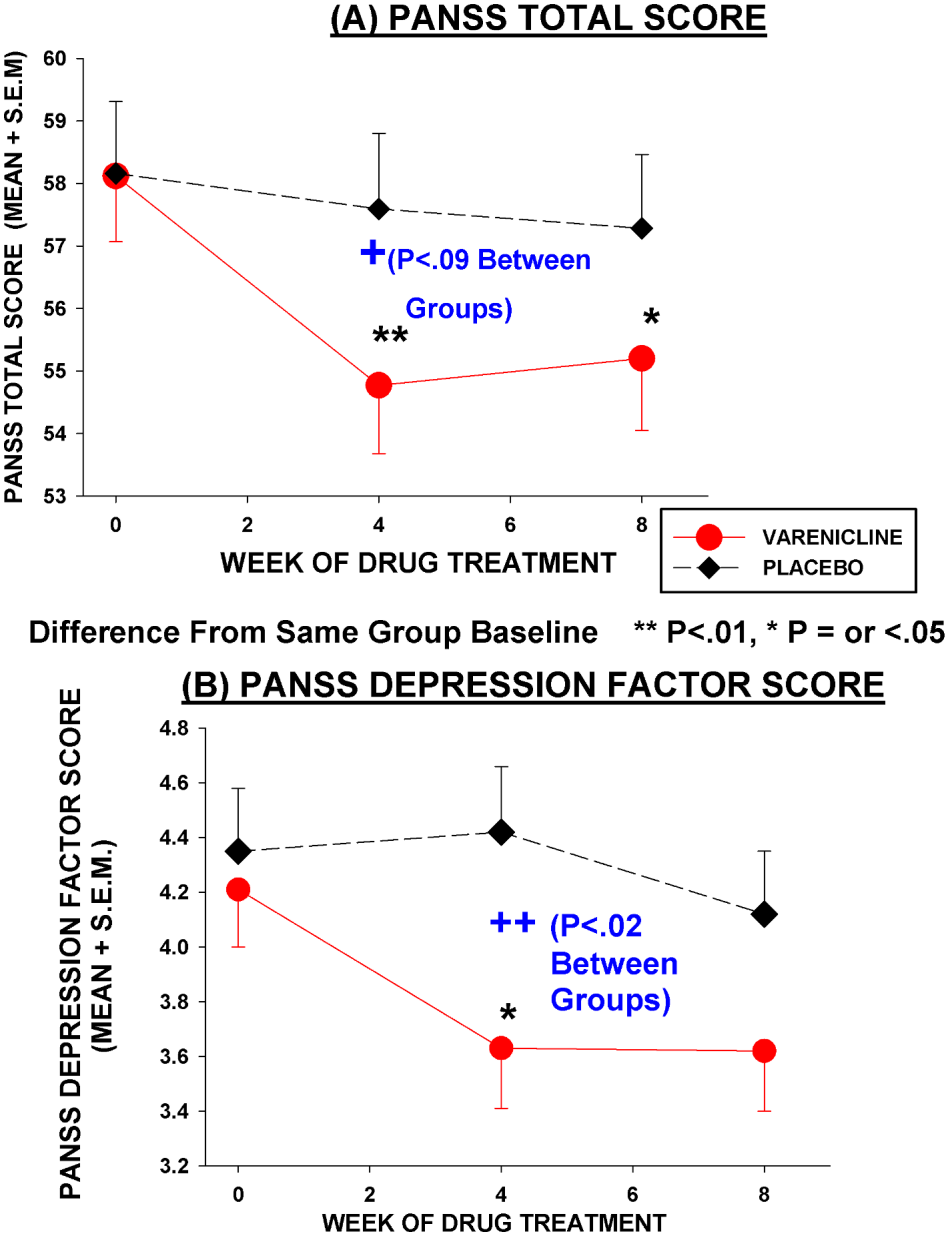
Effect of varenicline and placebo on PANSS Total and Depression Scores. N = 77, Varenicline = 38, Placebo = 39. Each value represents model adjusted least square mean score ± s.e.m. for that week, from mixed model ANCOVA. Overall Drug Effect between varenicline and placebo: PANSS Total F = 2.11, DF = 1, 71, P = 0.151; PANSS Depression Factor F = 4.79, df = 1,71, P = 0.032. Significance of difference from same drug baseline in varenicline treated patients by least square means t-test: * P<0.05, **P<0.01.

Varenicline subjects did not show any worsening of psychopathology scores, including positive symptoms and depression (Table 4, Fig 4). Although statistical analyses correcting for multiple comparisons using the BH procedure, showed no effects of varenicline on PANSS or SANS scores, there were some positive effects of varenicline on depression and avolition when *traditional* significance levels (i.e. uncorrected for multiple comparisons) were examined. There was a significant effect of varenicline on producing a greater decrease in PANSS *Depression* Factor scores (at traditional significance level P = 0.023), with the effect strongest at 4 weeks into treatment. The decrease in PANSS *Depression* Factor scores at week 4 was 13–16% (depending on whether calculations were based on adjusted or unadjusted scores) and effect size at 4 weeks was moderate (Cohen's d = 0.57). The decrease in PANSS *Depression* scores was also statistically significant (P<0.05) in the LOCF model and in a non-parametric test of observed cases at week 4 PANSS ratings. However, Calgary Depression Scale scores showed no difference between varenicline and placebo, with both groups showing small but statistically significant decreases. In weekly brief interviews to check on any depressive or other symptoms and emergent side-effects, only one varenicline patient expressed some increased feelings of depression and vague idea of hurting himself in week 3 of treatment. This patient did not express these symptoms in either week 2 or subsequent week 4, and his rating scale scores from week 4 vs. baseline did not show an increase in depression or psychosis. Varenicline treated patients showed a significantly greater decrease in the SANS Avolition sub-scores at 8 weeks than did placebo treated patients (traditional significance level P = 0.026) (Table 4), with a moderate effect size (partial eta square = .086). None of the SANS differences remained significance (at α = 0.05) at BH corrected significance levels. (See Table 4 for other trends.)

**Table 4:** Change from baseline in psychiatric symptoms scores with varenicline or placebo. Each value represents mean ± s.e.m. of model estimated difference score (wk_i_-baseline). SANS Modified Total = Sum of scores of Affective Flattering, Alogia, Avolition, Anhedonia. PANSS Depression is sum of scores from items G2+G3+G6 (adopted from Lancon [58]. F represents results of analysis of covariance, with baseline scores as covariate. PANSS and Calgary Depression scores used SAS Mixed Model with difference scores from week 4 and week 8 and baseline score as covariate. SANS was analyzed by SPSS Univariate ANCOVA GLM of week 8-baseline difference score with baseline score as covariate. Statistical significance of mean difference from 0 (i.e., no change) for measure for each drug group at specific time point:

Measure and Week of Study Drug Treatment	Varenicline	Placebo	T -test specific time point	Overall *Drug Effect* (F)	Overall *Drug x Time Effect* (F)
**PANSS SCORES (N = 77)**					
**PANSS TOTAL**					
Week 4	-3.56 ± 1.29**	-0.86 ± 1.42	T = 1.40, DF = 53, P = 0.166	F = 1.95, DF = 1,71, P = 0.167	F = 0.12, DF = 1,53, P = 0.732
Week 8	-3.05 ± 1.35*	-0.97 ± 1.40	T = 1.07, DF = 53, P = 0.289		
**PANSS POSITIVE**					
Week 4	-0.54 ± 0.50	-0.62 ± 0.54	T = 0.11, DF = 54, P = 0.917	F = 0.43, DF = 1,71, P = 0.514	F = 0.91, DF = 1,54, P = 0.343
Week 8	-0.23 ± 0.52	-1.01 ± 0.54	T = 1.03, DF = 54, P = 0.307		
**PANSS NEGATIVE**					
WEEK 4	+0.06 ± 0.61	+0.45 ± 0.67	T = 0.43, DF = 56, P = 0.670	F = 1.72, DF = 1,71, P = 0.195	F = 2.46, DF = 1,56, P = 0.122
WEEK 8	-0.52 ± 0.63	+1.19 ± 0.65	T = 1.89, DF = 56, P = 0.065		
**PANSS GENERAL**					
WEEK 4	-2.69 ± 0.79**	-0.80 ± 0.86	T = 1.62, DF = 56, P = 0.111	F = 1.31, DF = 1,71, P = 0.256	F = 1.56, DF = 1,56, P = 0.217
WEEK 8	-1.99 ± 0.83*	-1.54 ± 0.84	T = 0.38, DF = 56, P = 0.705		
**PANSS DEPRESSION**					
Week 4	-0.67 ± 0.24**	+0.23 ± 0.26	**T = 2.49, DF = 56, P = 0.016**	**F = 5.37, DF = 1,71, P = 0.023**	F = 0.86, DF = 1,55, P = 0.359
Week 8	-0.67 ± 0.25**	-0.10 ± 0.26	T = 1.58, DF = 56, P = 0.120		
**CALGARY DEPRESSION SCALE (N = 74)**					
Week 4	-0.63 ± 0.24*	-0.72 ± 0.27**	T = 0.25, DF = 53, P = 0.803	F = 0.08, DF = 1,67, P = 0.781	F = 0.00, DF = 1,53, P = 0.991
Week 8	-0.79 ± 0.26**	-0.88 ± 0.26**	T = 0.24, DF = 53, P = 0.815		
**SANS SCALE (N = 64)**					
**SANS Modified Total Score** Week 8 vs. Baseline	-4.66 ± 2.23*	-2.11 ± 2.30	NA	F = 0.633, DF = 1, 55, P = 0.430	NA
**SANS Avolition Sub-Score** Week 8 vs. Baseline	-1.48 ± 0.39*	-0.20 ± 0.40	NA	**F = 5.203, DF = 1,55, P = 0.026**	NA

* P<0.05,

**P<0.01.

### Side-Effects

There was no difference in change from baseline values in side-effects in the varenicline group when compared to the placebo group at any time point (see Tables in S2 File). This was true for total side effect score, nausea and vomiting side-effects, and other specific side effects. The results were similar in the mixed model analysis of difference scores for total side effects, and non-parametric analyses of difference scores for specific side effects. However when baseline side effects were not corrected for, varenicline subjects showed a higher percent of at least one report of nausea and vomiting compared to placebo subjects. No subject reported a clear increase of suicidal ideation and no suicides or new emergency acute depressive episodes occurred. No subject reported disturbing abnormal dreams and there was no difference in insomnia. Furthermore, upon review of several adverse events submitted to the IRB, it was concluded that no adverse event involving emergent psychiatric symptoms could be definitely or probably attributed to varenicline treatment.

## Discussion

The results of this study show that varenicline is a safe and effective medication for reducing cigarette smoking in schizophrenia, even in patients who do not indicate a definite desire to quit smoking, but it does not improve cognitive function in patients with chronic schizophrenia who were smokers and were being treated with a broad range of antipsychotics and other adjunctive treatments.

### Effects on Cigarette Smoking

The effects of varenicline on reducing cigarette smoking in this study are consistent with other studies showing that it is an effective medication for reducing smoking or assisting in smoking cessation treatment in non-psychotic smokers [1, 34] and in patients with schizophrenia [17, 35–38]. Our results suggest that if varenicline is going to be effective for reducing cigarette smoking in patients with schizophrenia then the effects should start to become evident in the first few weeks of treatment. It was not surprising that varenicline compared to placebo did not significantly increase cigarette smoking quit rates in this sample of patients with schizophrenia who did not express a desire to quit smoking, since almost all the studies which have found an effect of varenicline on abstinence rates have enrolled patients who wanted to quit smoking and had a quit date as part of the protocol. It is also possible that the lack of difference in quit rates between varenicline and placebo groups, could be due, in part, to the small beneficial effects of the weekly behavioral counseling on reducing smoking and motivating quitting in the placebo group. Furthermore, it is possible that with a much larger sample size, the approximately 1.5–2 times difference in quit rates in the varenicline vs. placebo group might have become statistically significant. Two other studies that enrolled patients with schizophrenia who wanted to quit smoking, and had a quit day as part of the study design, showed stronger effects [35],[36]. And a recent study [37] showed a high rate of maintenance of smoking cessation (45%) in schizophrenic smokers motivated to quit who were continually treated with varenicline for more than a year. Another study of varenicline which enrolled patients with schizophrenia who were not specifically selected on their desire to quit smoking [19], similar to our study, also noted that none of the patients quit smoking, although there were decreases in the number of cigarettes smoked and decrease in CO measures in the varenicline group.

### Effects on Cognition

The failure to show an effect of varenicline on improving scores on the MATRICS consensus battery, suggests that this drug may not be efficacious as a general cognitive enhancer in patients with schizophrenia. The lack of effect on improvement in the MCCB score were not due to a floor effect in patients with minimal cognitive impairment, since our baseline scores showed that most patients had moderate to severe cognitive deficits. Our results are consistent with the main results of neuropsychological tests in several other recent studies of the effects of varenicline in patients with schizophrenia [18, 19, 39]. Hong and associates [18] reported no significant differences in the Composite or Domain scores of the MATRICS battery or the Digit Symbol Substitution Task or the Connors CPT in schizophrenic patients treated with varenicline 1mg/day. Shim and associates [19], who studied both schizophrenic smokers and non-smokers, reported no differences between varenicline and placebo groups comparing baseline scores to week 8 scores in a sample of Korean schizophrenic patients. Roh and colleagues[39] reported no differences between varenicline and placebo on CPT, Stroop, n-Back and visual spatial memory tests in patients with schizophrenia. Some psychophysiological measures relating to stimulus or information gating, startle response, and saccadic eye movements are hypothesized to be related to underlying core neurophysiological deficits in schizophrenia. In the Hong et al. study [18], these measures were improved by varenicline. Studies in non-psychotic smokers [40] and in smokers with schizophrenia [41, 42] provide evidence to support the idea that varenicline may counter some of the neurocognitive deficits that smokers experience during the early phase of smoking abstinence. In one study when varenicline was compared to placebo there were significantly decreased deficits on the Digit Span Forward during abstinence, and in another study varenicline significantly attenuated the deficits in spatial working memory directly after acute smoking abstinence. It is therefore possible that if the effects of varenicline were studied after acute cigarette abstinence some stronger positive cognitive effects might have been found in our patients.

We cannot fully explain the improvement in MCCB scores in a sub-set of placebo patients who had complete MCCB data. It may be due to a practice effect although most components to the MCCB battery have been reported to show no or small practice effects on repeat testing [43, 44].

The lack of efficacy of varenicline as a general cognitive enhancer in schizophrenia may be related to its receptor pharmacology. Most of the nicotinic agents which have been recently tried as pro-cognitive agents for schizophrenia have been primarily α_**7**_—nicotinic agonists, partial agonists, or modulators, and in clinical trials some, but not all, of these compounds have shown positive cognitive effects in schizophrenia [45]. Although varenicline is a full agonist at α_**7**_ neuronal nicotinic receptors, it has relatively low affinity; it has much higher affinity as a partial agonist at α_2_β_4_ nicotinic receptors [3, 46–48]. The high affinity partial agonist effects at the α_2_β_4_ (EC_50_ 2.1–2.3 nM) receptors are associated with it stimulating dopamine release, which may compensate for nicotine's action of phasic dopamine release in the ventral tegmental region associated with cigarette smoking. Although a full agonist at the α_**7**_ receptor, because of its lower binding affinity, varenicline may have 24 times less functional potency at this receptor compared to the α_2_β_4_ receptor[3]. This difference in affinity and functional potency at the different receptors may be one of the factors involved the explanation the varenciline’s general efficacy as an anti-smoking agent and lack of clear efficacy as a general cognitive enhancer in schizophrenia, except in special acute abstinence conditions.

### Psychiatric Symptoms and Side-Effects

A few early published case reports[49, 50] and Med Watch case reports to the FDA, suggested that varenicline might have the potential to increase depression, psychosis, and suicide in patients with schizophrenia or other vulnerable patients. This has not been confirmed in results from large epidemiological studies of varenicline in clinical treatment as well as pooled analysis of clinical trials in non-psychiatric populations [51–54]. This conclusion is buttressed by the recent large scale re-analysis and review of psychiatric and other side-effects in controlled and observational studies of varenicline treatment reported by Gibbons and Mann [55] which found no increase in psychaitric adverse events attritubable to varenicline. In the current study no increase was found in any component of psychiatric symptoms in schizophrenic patients treated with varenicline, and there were either trends for decrease, or statistically significant decreases, in most symptom measures. Our findings that there was no increase in psychiatric symptoms with varenicline is consistent with the findings of three other recent double-blind studies of varenicline treatment in patients with schizophrenia [18, 19, 36], and open label studies or reports [17, 35, 38] which also reported no change or selective decrease in psychiatric symptoms including depression, psychosis, and negative symptoms.

A potential negative effect of varenicline cannot be excluded in acutely psychotic, or severely depressed or suicidal patients with schizophrenia, since our study, and several other double-blind studies, excluded these patients. However, the Gibbons study [55] reported no differences in rates of psychiatric adverse events in varenicline treated subjects who had a psychiatric event or suicide attempt in the prior year vs. those who didn't. Furthermore, a study in depressed patients found that varenicline decreased their depression rating scale scores [56] and another study showed no difference in depression or suicide in patients treated with varenicline who had past or current depression [57].

### Limitations

Limitations of the study, or factors affecting interpretation of some of the results, include the lack of testing for inter-rater reliability testing between sites, the lack of established norms for MATRCIS battery T-scores at the Israeli and China sites, the combination of inpatients and outpatients, and a sample of patients who were did not express a definite desire to quit cigarette smoking and had no quit date as part of the study design. Benzodiazepines can have small but significant effects on decreasing cognitive performance and 24% of our patients were receiving benzodiazepines, which could potentially affect their cognitive performance on the MATRICS battery. However, there was no difference in the percent of patients on benzodiazepines in the varenicline and placebo groups. Other limitations include the fact that the sample consistently of primarily male schizophrenic patients, with a small number of female subjects, and we could not confidently address whether there is a differential sex effect on any of our outcome measures. The multiple different antipsychotic medications these patients were on, although reflective of real world treatment, might have contributed to more “noise” and made it more difficult to detect an effect of varenicline on cognition. The very low baseline scores on the CDSS may have made it difficult to detect any effects of varenicline on improving depression on this measure, because of a ceiling effect. The limited behavioral counseling program involving 5–10 minutes sessions, is shorter than the 60 minute sessions used in some other studies, and this may have reduced potential abstinence effects or even greater smoking reduction.

## Conclusions

Our study showed that varenicline was effective for reducing cigarette smoking and smoking urges in patients with schizophrenia who were not strongly motivated to quit smoking. It was not a cognitive enhancer. It did not worsen any psychiatric symptoms. It may have a weak effect on improving some measures of depression or components of negative symptoms based on the significant effects on some of these measures with standard significance levels uncorrected for multiple comparisons, although a robust effect did not persist with BH corrected significance levels.

## Supporting Information

S1 File Additional Supporting Data for Methods Section. (DOC)

S2 File Side Effect Tables. (DOCX)

S3 File Data Files of Original Data compressed zip file. One excel file and 4 SPSS system files, total.
(SAV)

S1 CONSORT Checklist Consort Check List. (DOC)

S1 Protocol Varenicline Protocol Approved by IRB. (DOC)
