# Supplementary material for: Varenicline Effects on Smoking, Cognition, and Psychiatric Symptoms in Schizophrenia: A Double-Blind Randomized Trial
Source: PLoS One. 2016 Jan 5;11(1):e0143490. doi: 10.1371/journal.pone.0143490 (PMC4701439; doi:10.1371/journal.pone.0143490)
Supplement: S1 File — (DOC) [file pone.0143490.s001.doc]

**Detailed Inclusion and Exclusion Criteria For Subjects In Study And Additional Information On Statistical And Other Analysis Methods**

**Varenicline Effects On Smoking, Cognition, And Psychiatric Symptoms In Schizophrenia**

***1. Detailed Inclusion and Exclusion Criteria***

***Inclusion criteria***: Patients were male or female, 18-65 years of age, with a DSM-IV diagnosis of schizophrenia or schizoaffective disorder determined by best estimate diagnostic approach using research DSM-IV criteria diagnostic checklist, review of chart history and data from structured interviews. Patients were currently treated with antipsychotic medications, and judged to be clinically stable in symptom picture, and in a chronic and not a newly acute phase of their illness. Patients were current cigarettes smokers, who smoked at least 6 cigarettes a day for the past 6 months, or if currently hospitalized in a non-smoking facility, were regular smokers before hospitalization and had violated non-smoking rules in the hospital by smoking cigarettes on at least one occasion. Most of the inpatients came from the China site where inpatients were allowed to smoke cigarettes. 6six inpatients participating in US sites were in non-smoking facilities. Patients did not have to express a strong desire to quit smoking, but they were willing to try a drug which might decrease their smoking and might affect or improve their cognitive function. (They were informed during the consent process that they would receive either the varenicline drug or a placebo.) Patients had at least a minimal level of cognitive impairment, defined by a total score of ≤90 on the Repeatable Battery for the Assessment of Neuropsychological Status [RBANS]. Patients were on a stable dose of antipsychotic medication which had not changed in the last month.

***Exclusion criteria***: Subjects were excluded if they had a total PANSS score >90, had a PANSS depression item score >5, had a Calgary depression scale score >20, or had expressed definite suicidal ideation in the last month, or had made a documented suicide attempt or clinically serious documented suicide gesture in the last year. Patients with acute exacerbations of their psychosis in the last month which are judged to be clinically significant were excluded. Subjects who were current cocaine-crack, PCP, or methamphetamine abusers in the last month were excluded. Patients with a current neurological CNS disorder such as seizure disorder or stroke in the last year, who were currently receiving medications specifically for these conditions, were excluded. Women of childbearing age who were not using regular birth control were excluded. Patients with acute worsening of their psychosis in the last month which were judged to be clinically significant, and had not re-stabilized, were excluded. Patients with history of possible Downs syndrome, severe mental retardation, or more severe dementia (MMS<20) were be excluded. Patients with a documented history of a myocardial infarct, history of atrial arrhythmias or fibrillation with the last year, or a documented medical history of thromboembolic events were excluded. Subjects who had significant renal impairment (creatinine ≥ 1.5) were excluded.

**2. Methods - Additional Details**

**Randomization and Blinding**

Randomization was generated from computer generated randomization tables and the site of the principal investigator. Randomization assignment was in group of 4 with 2 active and 2 placebo assignments for each of 4 consecutive subjects randomized at a site. Randomization in blocks of 4 was done, s so that if there were any changes in subjects flow or research assistants during the course of the study at each site, the changes would be likely to equally affect subjects assigned to active and placebo administration at that site. A research pharmacist at each site assigned subjects to active or placebo, and prepared identical pills of either varenicline or matched placebo in weekly bottles labeled with subjects name and study number. Subjects were enrolled by the principal investigators and their research assistants at each site following subject inclusion and exclusion criteria described above. Both subjects and research evaluators as well as their treating psychiatrists were blind to the subject’s drug assignment thought the period of each subject’s participation. After completion of the subject’s participation and entry of the subject’s data into spreadsheets, the pharmacists sent sealed envelopes to the site investigator revealing the blinded assignment, so that data could be accurately assigned for active drug or placebo in spreadsheets.

**Sample Size Calculations and Study Recruitment**

Sample size calculations was originally based on estimates of sample size needed for data on the MATRICS battery, which was considered the most important outcome by the foundation, Stanley Research Foundation, which funded the major parts of the study. Based on several estimates of different percents improvements on the MATRICS battery score, using a power analysis program N query 3, we estimated a sample size of 20-30 in each grup would be sufficient to give 80% power to detect a difference a P=.05. Based on the higher estimate, this required 60 patients who would have evaluable data on MATRICS battery at baseline and 8 week of drug treatment testing. Because of drop outs during the study of subjects who dropped out before completing their second MATRICS battery, we increased subject recruitment-consent goals to 90-95 subjects, to obtain about 60 subjects with evaluable data on MATRCIS battery. Recruitment was completed when we had obtained over 60 subjects for whom had data on MATRCIS battery at baseline and drug treatment time point, although only 54 had complete enough MATRICS data for computation of overall Composite score. Around the time of termination of recruitment, changes in NY state regulations, banning smoking in outpatient residences and other outpatient related facilities controlled by New York State made it more difficult to continue to recruit patients with schizophrenia who were still regular smokers smoking at least 6 cigarettes/day.

***Statistical Analysis***

The main analysis for most variables was a mixed-model analysis of covariance (baseline value as covariate) using the SAS mixed procedure program with either unstructured or autoregressive (ar1) correlation structure, with *Drug* and *Site* as factors *Time* the repeated measure. Analyses were performed on the weekly values and separately on the difference scores from baseline for that week. For variables which deviated markedly from a normal distribution, transformations were attempted with log or square-root transforms, and statistical analyses were performed on transformed values when they provided a better approximation to a normal distribution. For variables whose distribution showed a significant difference from normality by Kolmogorov-Smirnov tests (SPSS Explore), additional analyses on observed cases at each time point were performed by non-parametric methods (Mann-Whitney U, Wilcoxon). For those variables which showed a markedly Poisson distribution even after transformation, the non-parametric analyses were used as the primary analysis.

**MATRICS Score calculations**.

For the MATRICS battery, T-scores and Domain and Composite scores were calculated with the revised MATRICS battery program (beta version supplied by Dr. Michael Green, UCLA) which allows calculation of some domain and composite scores with missing data on some tests or non-inclusion of social cognition module. We had not included the Social Cognition module in our MATRICS battery tests because some researchers had reported these responses to be unreliable in many patients with chronic schizophrenia..

However, some patients had missing data on one or more MATRICS tests which did not allow calculation of total Composite Score for that subject, but had sufficient data for calculation of some of the domain scores. This explains the different N’s for various domain and composite scores in Table 3. Figure 4 presents data from only those subjects who had complete data with Composite scores in the varenicline and placebo groups.

Because benzodiapines can have small effects on decreasing cognitive performance on some neuropsychologist tests, MATRICS battery was not performed on a day that a subject had received an acute prn dose of a benzodiazepines (usually for agitation) during the previous day.

**Nicotine and Cotinine Assay of Samples for Subjects From Beijing China** Site. (Assay performed at the Department of Biological Psychiatry, Shanghai Mental Health Center.)

1. Instruments And Chromatographic Conditions

The LC/MS analysis was performed on the Agilent 6430 Triple Quadrupole LC/MS System. A Zorbax C18 column (1.8µm, 2.1×50mm) was used for the chromatographic separation. The Mobile phase was 30% 10mM ammonium acetate (pH=4.0, adjusted by formic acid) versus 70% acetonitrile. The flow rate was 0.2ml/min and the injection volume was 5µl. The mass spectrometer was operated in positive ESI mode with the following parameters: 350 ◦C Gas temperature, 10 L/min gas flow, 4.0 kV ionization voltage in positive mode, 35 psi. The ion pairs m/z for cotinine: 177.1/80.1 and cotinine-d_3_ 180.2/80.1. nicotine 163.2/130.1 and nicotine-d_4_ 167.2/84.1.

2. Sample Preparation

Samples were prepared according to the methods described in the references [1-4] and adjusted accordingly. 0.5 ml of each plasma sample, QC sample or calibrator was combined with 40µl the internal standard working solution. And 50µl sodium acetate (2M) and vortexed for 10s. 4ml mixture of hexane: dichloromethane : isopropanol (65:32:3), vortexed 1min, and then concentrated 10min (3000r/min), the supernatant was evaporated to dryness at 30℃ with nitrogen. Samples were reconstituted in 125µl methanol for chromatography.

3. Calibration

Linearity was evaluated by analyzing taboo-free human serum to which nicotine cotinine, cotinine-d_3_ and nicotine-d_4_ had been added at six points for nicotine and seven points for cotinine. The linearity range for cotinine is 3.75-600ng/ml and for nicotine 1.25-50ng/ml.

Reference

1. Simultaneous Analysis of Nicotine, Nicotine Metabolites, and Tobacco Alkaloids in Serum or Urine by Tandem Mass Spectrometry, with Clinically Relevant Metabolic Profiles. *Clinical Chemistry* 48:9 1460–1471 (2002).
