# Supplementary material for: Varenicline Effects on Smoking, Cognition, and Psychiatric Symptoms in Schizophrenia: A Double-Blind Randomized Trial
Source: PLoS One. 2016 Jan 5;11(1):e0143490. doi: 10.1371/journal.pone.0143490 (PMC4701439; doi:10.1371/journal.pone.0143490)
Supplement: S2 File — (DOCX) [file pone.0143490.s002.docx]

**S3 Side-Effect Tables**

**Table S3-1 - Change from Baseline In Total Side Effects Scores**

| **Measure And Week Of Study Drug Treatment** | **Varenicline** | **Placebo** | **T-Test, Specific Time Point** | ***Drug Effect* Overall Model (F)** | ***Drug X Time Effect* Overall Model (F)** |
| --- | --- | --- | --- | --- | --- |
| **Difference Score in Total Side Effects (N=84)** | | | | | |
| Week 2 | -0.84 ± 0.76 | -1.37 ± 0.73 | T=0.74, P=0.64 | F=0.09, DF=1,78, P=0.770 | F=0.54, DF =2,118, P=0.5852 |
| Week 4 | -1.04 ± 0.75 | -1.70 ± 0.82* | T=0.59, P=0.55 |  |  |
| Week 8 | -0.90 ± 0.80 | -0.5+ ± 0.82 | T=0.29, P=0.77 |  |  |

Each value represents mean ± s.e.m of model estimated difference score ( wk**_i_**-baseline). Difference of mean from 0 (no change) for measure for each drug group at specific time point: * P<.05. F based on mixed model analyses of difference values with baseline covariate including all weeks of data.

**Table S3-2 - Percent Of Patients Reporting Specific Side Effect At Any Time During 8 Week Of Study Drug Treatment**

| Side Effect | Placebo | Varenicline | Significance of Difference |
| --- | --- | --- | --- |
| Nausea | 18 | 43 | ᵪ2 _= 6.23, P=.013_ |
| Vomiting | 11 | 30 | ᵪ2 _= 4.72, P=.030_ |
| Insomnia | 24 | 13 | ᵪ2 _= 1.98,P=.160_ |
| Dizzy | 29 | 25 | ᵪ2 _= 0.16,P=.687_ |
| Restless | 36 | 25 | ᵪ2 _= 1.11, P=.292_ |
| Headache | 20 | 13 | ᵪ2 _=0.87, P=.352_ |
| Sedation | 38 | 33 | ᵪ2 _=0.26, P=.611_ |
| Malaise | 31 | 43 | ᵪ2 _=1.19, P=.276_ |
| Rash | 13 | 15 | ᵪ2 _=0.05, P=.826_ |

Each number represents percent of patients who reported an occurrence of the specific side effect, at a “mild” level or greater, at least once during the 8 weeks of study drug treatment.

**Table S3-3 - Difference In Side Effects Scores Of Combined Nausea And Vomiting For Specific Week From Baseline Side-Effects Before Start of Study Drug**

| **Week of Study** | **Varenicline**  (N’s=22-40) | **Placebo**  (N’s=27-45) | **Non - Parametric Test** |
| --- | --- | --- | --- |
| Week2 Mean  (N=85) Median | 0.08 ± 1.46  0.00 | -0.13 ± 0.94  0.00 | Z=-0.901, P=0.368 |
| Week 4 Mean  (N=74) Median | 0.00 ± 1.27  0.00 | -0.14 ± 0.68  0.00 | Z=-0.712, P=0.477 |
| Week 8 Mean  (N=65) Median | -0.13 ± 1.15  0.00 | -0.03 ± 0.84  0.00 | Z=-0.581, P=0.561 |
| Week 12 Mean  (N=49) Median | -0.14 ± 1.58  0.00 | -0.11 ± 0.85  0.00 | Z=-0.107, P=0.915 |

Each number represents either Mean ± S.D. or Median of sum of side effects score of nausea + vomiting for that week minus the baseline score. Because difference score were not normally distributed, non- paramedic test Mann-Whitney U was used to determine significance of differences.
