# Supplementary material for: Varenicline Effects on Smoking, Cognition, and Psychiatric Symptoms in Schizophrenia: A Double-Blind Randomized Trial
Source: PLoS One. 2016 Jan 5;11(1):e0143490. doi: 10.1371/journal.pone.0143490 (PMC4701439; doi:10.1371/journal.pone.0143490)
Supplement: S1 Protocol — (DOC) [file pone.0143490.s005.doc]

**Varenicline as Treatment for Cognitive Deficits and Cigarette Smoking in Schizophrenia – Efficacy and Predictors**

**I BRIEF BACKGROUND AND RATIONALE-SUMMARY**

Most schizophrenic patients smoke cigarettes and many are heavy smokers and are very reluctant to stop smoking. There is some experimental evidence that administering nicotine to schizophrenic patients may improve some aspects of their cognitive function. There is also literature establishing abnormalities or deficits of the α_7_ nicotinic receptors associated in a complex way to schizophrenia. Varenicline is an α_7_ nicotinic agonist and we would predict that varenicline should benefit schizophrenia by improving cognitive function. It may have some of the cognitive benefits of nicotone administration and at the same time reduce cigarette smoking. If varenicline showed cognitive enhancement properties in patients with cognitive deficits in schizophrenia, this would be an important breakthrough in improving the longer term functional outcome of many patients with this illness. This provides a strong reason for conducting a careful study to evaluate whether adding varenicline to the medication of patients who are treated with standard antipsychotic medication will significantly improve their cognitive function. We also wish to tie in any beneficial effect of this drug to the abnormalities in reelin, GAD_67_, and DMNT1 mRNA in schizophrenia presented in the work of Costa, Guidotti, and Davis and their collaborators.

**II RESEARCH PLAN**

***2.1 General Overview.***

This will be an 8 week parallel group placebo controlled double-blind experimental design in which cognitive function and psychopathology will be evaluated in schizophrenics (DSM IV diagnoses of schizophrenia or schizoaffective disorder). (Extension to 12 weeks at some sites including NKI site for smoking and psychopathology measures.) Patients will be tested on the MATRICS cognitive battery. Psychiatric status will be evaluated with the PANSS scale. Depression and any emerging suicidal ideation will be assessed by the CALGARY Depression scale. Negative symptoms for alogia and anhedonia will be assessed at beginning and end by abbreviated sections of the SANS scale. Changes in methylating enzyme functions related to GABAergic function will be assessed by measuring DNMT1 and GAD67 mRNAs in lymphocytes. These measures will be correlated with cognitive changes produced by varenicline. Because many schizophrenic patients are chronic cigarette smokers, and varenicline also affects cigarette smoking, cigarette smoking will be monitored by smoking questionnaires and nicotine and cotinine in plasma.

***2.2 Patients***

***Number of Patients***. Up to 100 patients will be enrolled in the study and we expect a 10-20% drop out rate. Valid cases for analysis of each variable will be considered subjects who have completed at least 1 post-baseline evaluation on that variable.

***Inclusion Criteria***: Patients will be male or female, 18-65 years of age, with a DSM-IV diagnosis of schizophrenia or schizoaffective disorder determined by best estimate diagnostic approach using research DSM-IV criteria diagnostic checklist, review of chart history and data from structured interviews. Patients will be currently treated with antipsychotic medications, and be judged to have a clinically stable symptom picture and not currently in actue exacerbation of their psychotic symptoms. Patients will have a minimal level of cognitive impairment; defined by a total score of ≤90 on the Repeatable Battery for the Assessment of Neuropsychological Status [RBANS] (The total score for normal subjects is mean±sd 100±15). All schizophrenic patients will either be current cigarette smokers or patients who were regular cigarette smokers in the past, and are not currently smoking daily because they are hospitalized in a non-smoking facility.

***Exclusion criteria***: Subjects will be excluded if they have a total PANSS score>80, have a PANSS depression item score >5, have a Calgary Depression score >20, have expressed definite suicidal ideation in the last month, or have made a documented suicide attempt or clinically serious documented suicide gesture in the last year. Subjects who have a history of multiple serious suicide attempts or clinically serious gestures in the past will be excluded. Subjects who meet DSM-IV criteria for current cocaine-crack, PCP, or methamphetamine abuse in the last month will be excluded. Patients who are currently smoking marijuana will be excluded. Patients with a current neurological CNS disorder such as seizure disorder or stroke in the last year, who are currently receiving medications specifically for these conditions, will be excluded. Patients with history of possible Downs syndrome, severe mental retardation, or dementia will be excluded. Subjects who have significant renal impairment (creatinine ≥ 1.5) will be excluded. Patients with acute exacerbations of their psychosis in the last month which are judged to be clinically significant, and have not re-stabilized, will be excluded. Patients with a documented history of a myocardial infarct, documented history of atrial arrhythmias or fibrillation within the last year, or a documented medical history of thromboembolic events will be excluded. Subjects who are currently treated with varenicline, at time of screening for the study, will not be enrolled in the study.

***2.3 Antipsychotic and Other Medication***

Patients will be treated with stable antipsychotic medication. They may be treated with accessory psychotropic medication (mood stabilizers, antidepressants, antianxiety agents, antiparkinson agents) if these medications have been regularly prescribed for at least 2 weeks before any study cognitive tests is performed. Prn doses of antipsychotic drugs are permitted they will be maintained on current drugs they are receiving which are used to treat medical conditions. Psychotropic and medical drugs will remain stable during the course of the study except for clinical conditions or serious emergent side-effects indicate that a change is immediately needed for clinical care.

***2.4 Study Medication***

After baseline evaluation the patient will receive study medication, active or placebo varenicline, for a period of 12 weeks. Varenicline will be administered in a dose of 0.5 mg - 1.0 mg/day for the first week, and 2.0 mg/day dose (two 1 mg tablets) for the remainder of the study period. If the patient experiences persistent nausea or other side-effects which he does not become tolerant to over the course of 2-3 weeks to the dose can be reduced to 1 mg/day.

***2.5 Psychiatric Evaluations***

Patients will be evaluated with PANSS rating scale, Calgary Depression Scale at baseline, and then monthly and end of study. The modified SANS scale will be administered at baseline and end of study. Patients will be seen and evaluated with a brief interview at least weekly by research staff team. If the patient’s psychiatric status appears to be significantly worsening, or if he expresses a clinically significant increase in depression or suicidal ideation, a PANSS, and Calgary Depression scale will be repeated more frequently.

***2.6 Cognitive evaluations***

A) The patient will be evaluated with the RBANS to see if he qualifies on cognitive scores as defined in inclusion criteria. He will be given a brief trial on the Matrix mazes to reduced practice effects on this test.

B) Patients who qualify on the basis of RBANS scores will be administered the MATRICS Cognitive battery at baseline and last month of study. The MATRICS Battery is an expert consensus cognitive battery which has been developed to be used in studies of psychoactive drugs that may benefit cognition in schizophrenia. The Social Cognition MSCEIT will be excluded because of previously documented difficulty in administering this to seriously ill schizophrenic patients and questions about its usefulness and validity.

C) At the U.S. sites another measure of spatial memory was added, and computer navigation task ( 'water task') which is a human analogue of a spatial memory task sued in many animal studies.

***2.7 Side - effects***

Side-effects will be evaluated with a side-effects check list administered biweekly for the first month and then monthly and at end of study. Furthermore, at each weekly meeting the patient will be asked about nausea, vomiting, strange dreams, feelings of depression, and any suicidal ideation.

***2.12 Cigarette Smoking Assessments***

Since smoking is highly prevalent in schizophrenic patients, varenicline has been shown to have antismoking effects in normals and in our preliminary studies also in schizophrenic patients], and it is also possible that nicotine itself has some cognitive effects, we will assess the degree of cigarette smoking in our subjects. Some e Measures will be taken at baseline and last month of study, and others at baseline 4, 8 weeks, and last month of study.

***A). Cigarette Dependence***. To measure at baseline the degree of dependence in current cigarette smokers, we will use the *Cigarette Dependence Scale* (Ettner et al 2003), in an interviewer-administered version.

***B).*** ***Self-report interview assessment***. Self-report of number of cigarettes smoked per day over the last week. Measurements will be made at baseline, 4 weeks, and end of study

***C) Smoking Urges***- A short version Tiffany Smoking urges scale (Cox, Tiffany, Chirsten 2001)

***D)*** ***Nicotine and Cotinine in plasma***. Measurements of plasma nicotine and cotinine will be made in plasma using samples at baseline, week 4 and week 8. It with be analyzed in the Analytical Psychopharmacology Laboratory (Tom Cooper, director).

*Nicotine and Cotinine Assay*

Nicotine and cotinine are assayed in plasma in the Analytical Chemistry Laboratory at the Nathan Kline Institute for Psychiatric Research, director Tom Cooper. The assay is a GC/MS assay, following the procedure described in Davis (Davis1986) with modifications for GC/MS detection. The method involves liquid/liquid extraction with N - ethyl nornicotine and Nicotine D4 and Cotinine D3 as internal standards. Separation is achieved via gas chromatography with a Restek RtX-5 amine 15m 0.25ID 0.50 um df, crossbond 5% diphenyl 95% dimethylpolysiloxane column. Nicotine is run in PCI mode; target ion 163, cotinine is run in EI mode, target ion 98. The detection limit of accurate quantification of nicotine with this assay is 3 ng/ml with intra- and inter-coefficients of variation of 4.7 and 5.9%. Cotinine limit of detection is 5 ng/ml with intra- and inter-assay coefficient of variation of 3.4% and 5.2%.

***2.9 Neurochemical Measurements***

DNMT, TET, Glucorticoid receptor, BDNF, and related mRNA's will be assessed in lymphocyte samples draw at b baseline and week 8 of study.

***A) Preparation of Lymphocytes***

Lymphocytes will be isolated with the Ficoll-Paque Plus method and reagents using the Amersham Kit (Biosciences2001-2006). Defibrinated or anticoagulant-treated blood is layered on the Ficoll-Paque PLUS solution and centrifuged for a short period of time. Different migration during centrifugation results in the formation of layers containing different cell types. The bottom layer contains erythrocytes which have been aggregated by Ficoll, and, therefore, sediment completely through the Ficoll-Paque PLUS. The layer immediately above the erythrocyte layer contains mostly granulocytes which, at the osmotic pressure of the Ficoll-Paque PLUS, attain a density great enough to migrate through the Ficoll-Paque PLUS layer. Because of their lower density, the lymphocytes are found at the interface between the plasma and the Ficoll-Paque PLUS with other slowly sedimenting particles (platelets and monocytes). The lymphocytes are then recovered from the interface and subjected to short washing steps with a balanced salt solution to remove any platelets, Ficoll-Paque PLUS and plasma. Since this method can be adapted to very small volumes of blood, it is particularly suitable for isolation of lymphocytes where only limited quantities of blood are available.

**B) *RNA Extraction***

Total RNA from lymphocyte was isolated using the TRIzol reagent (Life Technologies 15596-026; Life Technologies Corporation, USA; Mannhalter et al., 2000) and further purified using the Qiagen RNeasy Kit (Qiagen, Valencia, CA, USA***C)***

***C) Real-time polymerase chain reaction (PCR) quantification***

Total RNA was converted to cDNA using the Applied Biosystems (USA) High Capacity Archive Kit (4368813). Relative quantitative real-time polymerase chain reaction (qPCR) was performed with the Applied Biosynthesis Real-Time PCR system using Fermenta Maxima SYBR Green/ROX qPCR Master Mix (K0222; Fermentas International Inc., Canada). PCR mixtures were run on a Stratagene (USA) Mx3005P QPCR System. Primers were designed to cross over one intron to amplify cDNA and yielding an amplicon of between 75-200 base pairs. Dissociation curves were conducted to establish the presence of a single amplicon at the predicted melting temperature and a lack of primer-dimer formation. A comparative threshold cycle (C_T_) validation experiment was done to determine target and reference primer efficiency. For normalization of mRNAs expression, β-actin and GAPDH were used as internal controls. For the respective housekeeping genes, we measured the gene stability using the NormFinder alogaithm ((Andersen CL, Jensen JL, Orntoft TF, Normalization of real-time quantitative reverse transcription-PCR data: a model-based variance estimation approach to identify genes suited for normalization, applied to bladder and colon cancer data sets, Cancer; 64:5245-5250, 2004), which allows the identification of the best and suitable housekeeping gene for data normalization. For the respective genes studied, our preliminary data showed similar results when the data was normalized to β-actin or GAPDH, and because β-actin had the highest housekeeping gene stability (NormFinder), we normalized our data to β-actin. C_T_ value was used for relative quantification of target gene expression and normalized to β-actin and the relative expression levels were calculated as C_T_ (Livak & Schmittgen, 2001, Schmittegen & Livak, 2008). **2**.

***2.10 Outline of Research Evaluation Schedule***

A) Pre- consent screening -Screening-Chart review of medical and psychiatric diagnosis, and psychopathology and behavior.

*B) Consent*- RBANS, PANSS, CALGARY DEPRESSION, Modified SANS Scale, smoking surges scales

*C)Baseline*- MATRICS Cognitive Battery, Cigarette Dependence Scale, Current Smoking questionnaire, Plasma Sample for Nicotine-Cotinine, Blood Sample for determination of α_7_ mRNA and DNMT1 and GAD_67_ mRNA’s in lymphocytes .Side-effect checklist.

DNA Sample may be collected at baseline or any later point if the subject consents to this additional procedure.

*D)Weekly*- weekly interview, with questioning of major changes in psychiatric status or side effects, drug distribution, counting of pills from previous week.

*E)Week 2*- side-effects checklist

*F)Week 4,8*- PANSS, Calgary Depression Scale, side effects checklist, nicotine-cotinine sample, smoking urges scale, side-effects checklist.

*G)Week 8-12* -PANSS, Calgary Depression Scale, side-effects checklist list, Blood Sample for determination of α_7_ mRNA and DNMT1 and GAD_67_ mRNA’s in lymphocytes, smoking urges scale , MATRICS Cognitive Battery, Modified SANS, Cigarette Dependence Scale, Current Smoking questionnaire, Plasma Sample of Nicotine Cotinine, side effects checklist.

***2.11*** ***Criteria for subject termination from study***

Subjects will be terminated from participation in the study if they develop severe nausea or vomiting which does not show tolerance over the first four weeks of treatment, or if they request discontinuation because of this side-effect. Subjects will also be terminated if their PANSS scores show a 35% or greater increase on two consecutive ratings, including at least a 30% increase in positive symptoms, and a CGI score of much worse. They will be also be terminated if the treating physician believes their clinical condition has gotten much worse and requests discontinuation. If the subjects Calgary Depression Scale score increases by >50% on two consecutive ratings, and sum is also > 16, the patient will be terminated from the study. They will also be discontinued if they have persistent suicidal ideation or make a suicidal attempt or gesture. If the patient exhibits a marked increase in psychotic or depressive symptoms from the previous week, which is considered immediately clinically significant and threatening to his life or continued functioning or requiring immediate rehospitalization, the patient will be terminated from the study. If he makes a serious suicidal gesture or attempt in the prior week or expresses definite suicidal intent he will be immediately terminated from the study. Furthermore, subjects have the right to withdraw consent at any time.

***2.12 Subject Payments***

Subjects who sign consent and complete the RBANS will be paid $15. Subjects included in the treatment trail will be paid $15 per week for each week that they cooperate with all standard procedures of the study. They will be paid an additional $10 for the baseline and end of study blood samples. They will be paid an additional $5 for completing a neuropsychological battery test session. For subjects who are not inpatients at MPC or residing on the grounds transitional residence will be paid up to $10 for each occasion they need to make special trips to a site for study research procedures in order to cover transportation costs. Subjects are paid $5 if they agree to the genetic sample. Payment levels for outpatients are slightly greater than for inpatients.

**III STATISTICAL ANALYSIS**

***3.1 General***

The primary dependent outcome variable, will be the average score on all tests of MATRICs battery (converted to T scores from different tests). Secondary cognitive analysis will be the of the hypothesized effects of attention-vigilance, verbal memory, and visual spatial memory. Secondary outcome variables will be cigarette smoking variables( cigarettes smoked, CO, nicotine and cotinine in plasma, smoking scores, Calgary depression scores).The general analysis approach will use a repeated measures analysis of variance. If there are appreciable missing data on measures with several ( >2) sequential testings a mixed model analysis will be used. If the data deviates significantly from normality, a non-parametric approach may be utilized using Wilcoxon matched pair’s signed-ranks, or Friedman’s two way analysis of variance. To examine whether there are differences in effects in patients with different degrees of cigarette smoking, we will compare smoking measures in patients assigned to placebo or active drug, and correlate effects on neuropsychological test changes with the smoking measures described above. Paired T-tests, or repeated measures ANOVA, will be used to analyze differences in the biochemical measures. Pearson or Spearman correlations will be used to examine the relationship between baseline neurochemical measures to cognitive tests performed at baseline, and both baseline neurochemical measure and change in these measures will be correlated with change in neuropsychological test scores. Additional analyses may use these neurochemical measures or changes during treatment as a covariate in an overall repeated measures analysis of variance.

***3.2 Power Analysis.***

It is difficult to do a power analysis using the MATRICS battery total score or sub-scores as outcome measures in the two groups (active vs placebo) because we have not utilized this battery in the past, and there is not substantial published data on changes with this cognitive measure in schizophrenic patients with a drug design similar to our own. We used N Query Advisor 3.0 to calculate hypothetical N’s required on various assumptions. Using an α=.05, and a two group univariate analysis of variance model, with a 18-28% improvement in group scores in the varenicline group relative to the placebo group, and N of 15 or 29 per group would have an 80% to detect differences. A sample size of 60 for both groups combined is at or above these estimates of group size needed from the above hypothetical analyses. Using specific cognitive tests (CPT or visual spatial dot measures) measures from previous experiments we conducted with Nicotine active and placebo spray in one study, and olanzapine vs haloperidol in another study, N Query Advisor 3.0 calculated group sizes needed for 80% confidence at 15-20 per group. Therefore, both these analyses suggest that a group size of 60 (30 per active and 30 per placebo group) is sufficient for our experimental design.

***Amendments to IRB Protocol***:

Summary of Amendments

*Amendment 1*: Modification of risk statements in consent form in response to discussion with FDA

*Amendment 2:* Addition of MATRICS Battery of neuropsychological test-

*Amendment 3*: Additional survey questions and proposed advertising flyer

Amendment 4: Minor changes in procedures; coordination with Stanley Grant; addition of inpatients.

*Amendment 5*: Addition of family history data and smell test (This part of protocol was not carried out extensively because of lack of funding for smell test battery).

*Amendment 6*: Approval of Israeli Collaborative Site.

*Amendment 7:* Approval of NKI collaborative site.

*Amendment 8*: Addition of Baseline/screening EKG test at start of study, minor changes in cardiac

Exclusion criteria and expansion to involve RPC inpatient subjects on CREF.

Amendment 9-*Spatial Memory Task In 'Controls*

The object of this amendment is to compare performance on the *Virtual Morris Water Maze Task*, a measure of spatial memory, memory task, that we have obtained dint he original main protocol, to normal controls, who will be the subject of this amendment.

Subjects:

The subjects for this sub study will be male and female controls, 21-65 years of age who do not have a diagnosis of schizophrenia or psychotic disorders, major depressive disorder, bipolar disorder, ADHD, autism spectrum disorder downs syndrome, or dementia or Alzheimer's disease. They will not be currently taking antipsychotic, antidepressant, amphetamines or ADHD medication or varenicline. They will not have a major neurological illness for which they are currently being treated with medication or electrical or prior surgical therapy. 15 subjects will be cigarette smokers and 15 subjects will be current non-smokers. Assuming a 10-15% drop out rate, to obtain 30 valid subjects we may enroll or consent up total of 35 subjects.

Subjects Recruitment:

Subjects will be recruited from NKI research outpatient clinic control pool and volunteers, students and staff members of Nathan Kline Institute and Rockland Psychiatric Center. If we do not find enough subjects from these sources and want to recruit from a larger pool from Rockland County through advertisements, we will submit an advertisement for approval to the NKI IRB.

Procedures:

1. The patient will have brief interview and fill out questionnaires to assessing his medical and psychiatric history, current treatments, medications, and cigarette smoking.

2. They will bee administered the RBANS neuropsychological battery. This is a paper and pencil short neurocognitive battery which takes about 20-30 minutes.

3. Take a computerized test, *Virtual Morris Water Maze Task,* in which they will move a pointer around a pool of water on the computer screen. This will take about a half hour or less.

(In Phase I patients had 4 hidden platform trial blocks of 3 trials each, with random placement of the platform in the four quadrants, and 60 seconds to locate the platform until the platform becomes visible by raising it above the surface of the water. Measures in Phase 1 were latency to finding the platform (seconds) and distance (path length/pool diameter). In Phase II there was one 45-second trial with the platform visible, and then a similar trial with the platform in the same location but removed from the visual environment).

No drug is administered in this study.
